# Health-Promoting Properties of Natural Flavonol Glycosides Isolated from *Staphylea pinnata* L.

**DOI:** 10.3390/ijms25115582

**Published:** 2024-05-21

**Authors:** Ida Paolillo, Giuseppina Roscigno, Michele Innangi, Jesús G. Zorrilla, Gianmarco Petraglia, Maria Teresa Russo, Federica Carraturo, Marco Guida, Alessandra Pollice, Alessio Cimmino, Marco Masi, Viola Calabrò

**Affiliations:** 1 Department of Biology, Complesso Universitario Monte Sant’Angelo, University of Naples Federico II, Via Cintia 4, 80126 Naples, Italy; ida.paolillo@unina.it (I.P.); giuseppina.roscigno@unina.it (G.R.); federica.carraturo@unina.it (F.C.); marco.guida@unina.it (M.G.); apollice@unina.it (A.P.); vcalabro@unina.it (V.C.); 2 EnviXLab, Department of Biosciences and Territory, University of Molise, Contrada Fonte Lappone, 86090 Pesche, Italy; michele.innangi@unimol.it; 3 Department of Chemical Sciences, Complesso Universitario Monte Sant’Angelo, University of Naples Federico II, Via Cintia 4, 80126 Napoli, Italy; jesus.zorrilla@uca.es (J.G.Z.); petraglia.gianmarco17@gmail.com (G.P.); mariateresa.russo2@unina.it (M.T.R.); alessio.cimmino@unina.it (A.C.); 4 Allelopathy Group, Department of Organic Chemistry, Facultad de Ciencias, Institute of Biomolecules (INBIO), University of Cadiz, C/Avenida República Saharaui, s/n, 11510 Puerto Real, Spain

**Keywords:** *Staphylea*, flavonoids, flavonol glycosides, antioxidants, antimicrobial, antiaging ingredients

## Abstract

*Staphylea*, also called bladdernuts, is a genus of plants belonging to the family Staphyleaceae, widespread in tropical or temperate climates of America, Europe, and the Far East. *Staphylea* spp. produce bioactive metabolites with antioxidant properties, including polyphenols which have not been completely investigated for their phytotherapeutic potential, even though they have a long history of use for food. Here, we report the isolation of six flavonol glycosides from the hydroalcoholic extract of aerial parts of *Staphylea pinnata* L., collected in Italy, using a solid-phase extraction technique. They were identified using spectroscopic, spectrometric, and optical methods as three quercetin and three isorhamnetin glycosides. Among the flavonol glycosides isolated, isoquercetin and quercetin malonyl glucoside showed powerful antioxidant, antimicrobial, and wound healing promoting activity and thus are valuable as antiaging ingredients for cosmeceutical applications and for therapeutic applications in skin wound repair.

## 1. Introduction

The exploration of poorly investigated plants for their natural bioactive components can lead to the discovery of new compounds with potential therapeutic benefits for human health. One of the specific uses of natural bio-derived compounds concerns their antioxidant potential [1,2,3]. Antioxidants help neutralize the free radicals, reducing the potential for damage to cells and tissues. The skin, in particular, is the major interface between the human body and its environment. UV radiation is absorbed by skin molecules and generates reactive oxygen species (ROS), causing “oxidative damage” to cellular components like cell walls, lipid membranes, mitochondria, and DNA. Oxidative stress is a major contributor to premature skin aging. Furthermore, plant-derived compounds, along with naturally sourced ingredients, show promise in accelerating wound healing, especially when incorporated into the cosmetic formulation [4].

**Figure 1:**
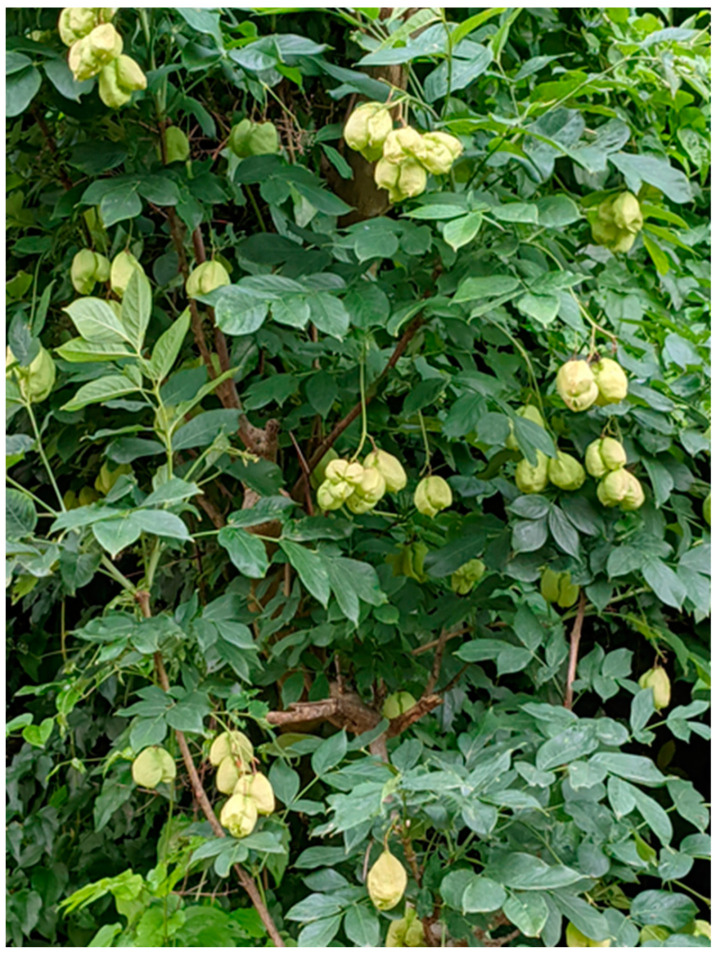
*Staphylea pinnata* plant.

*Staphylea pinnata* L., also referred to as the European bladdernut, is the only extant species of the genus Staphylea native to Central-Eastern Europe (Figure 1).

It belongs to the family Staphyleaceae and the order Crossomatales. The species has a distribution restricted to east-central Europe, ranging from France to Ukraine and extending eastward to the Transcaucasus region. The seeds are edible and are occasionally called the “false pistachio” because of their similarity in flavor to *Pistacia vera* L. The plant is fairly abundant in Italy, with a limited distribution. It grows at altitudes between 0 and 900 m in almost all regions of Italy except Valle d’Aosta, Liguria, Sardinia, and Sicily. This plant often inhabits calciphilous soils, commonly in moist woods. In the past, this species was utilized for wood-carving and other traditional purposes in Europe [5]. Native Americans used an infusion of *Staphylea trifolia* L. for its antirheumatic, dermatological, sedative, and gynecological properties [6]. The seeds gained popularity due to their aesthetically pleasing hue, shape, and long-lasting nature. The Celts utilized them to create diverse embellishments. Furthermore, the seeds of this plant are rich in fat and can serve as a valuable oil source. Previously, they were pulverized and used in animal feed since it was thought that they might promote robust health and extend the lifespan of livestock. Additionally, they were utilized as medicinal remedies for ailing youngsters, as they were thought to possess therapeutic properties, although, if taken in excessive amounts, they may induce vomiting [7].

Data from the literature on the chemical composition of the flowers and leaves have shown promising results, including notable cytotoxic and antibacterial properties [6,8,9,10,11]. *Staphylea* spp. produce bioactive metabolites with antioxidant properties, including polyphenols, but their nature remains undefined [6]. One study referred to the flavonoids isolated from *Staphylea bumalda* L. without, however, evaluating their bioactivity [12]. In 2019, a work was published regarding the bioactive components present in the seeds of *S. pinnata* [13], but to date, there is no detailed report regarding the chemical composition of the bioactive constituents present in the aerial parts of the plant.

Here, we report the isolation of six flavonol glycosides from the hydroalcoholic extracts of *S. pinnata*’s aerial parts collected in southern Italy. The six pure metabolites were tested for their antioxidant, antimicrobial, and wound healing-promoting activities in human keratinocytes in light of their application in the cosmeceutical industry.

## 2. Results

### 2.1. Extraction of S. pinnata’s Aerial Parts

**Figure 2:**
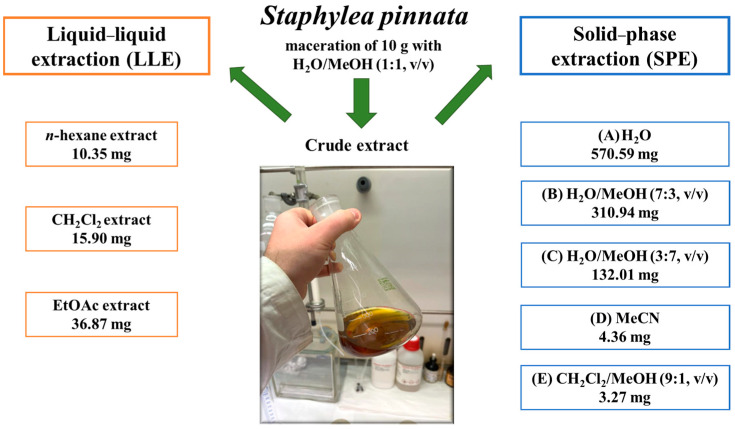
Extraction procedures applied to 10 g of the dried aerial parts of *S. pinnata*.

The dried aerial parts of *S. pinnata* were macerated using a hydroalcoholic solution (crude extract) and partially purified using two different methods, as detailed in Section 4. In particular, a liquid–liquid extraction (LLE) and a solid phase extraction (SPE) were carried out to obtain different organic extracts and fractions, as summarized in Figure 2.

Briefly, three organic solvents with increasing polarity were employed for the LLE, while the SPE was carried out via stepwise elution with different solvent mixtures. The extraction yield of the SPE was higher than the LLE and the chromatographic profile of all the fractions obtained was compared using TLC on a silica gel and reverse phase. The results showed different metabolite contents in the various fractions obtained, and in particular, some major compounds were observed in both the EtOAc extract of the LLE and the C fraction of the SPE.

### 2.2. Cytotoxicity and Antioxidant Properties of S. pinnata’s Crude Hydroalcoholic Extract and the LLE and SPE Fractions

**Figure 3:**
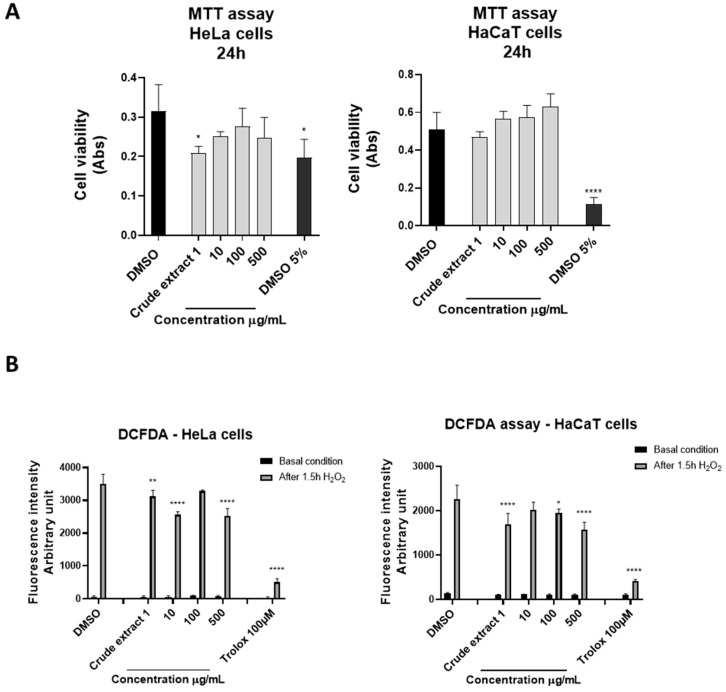
(**A**) MTT viability test. HeLa and HaCaT cells were incubated with the indicated amount of crude hydroalcoholic extract for 24 h. The values were the mean’s six values for each experimental point of two independent biological replicates. Each mean was compared using a Dunnett’s multiple comparisons test of a one-way ANOVA (*p*-value * *p* < 0.01, **** *p* < 0.0001). (**B**) DCFDA assay. HeLa and HaCaT cells were seeded and pre-treated for 4 h with 1, 10, 100, and 500 μg/mL of crude hydroalcoholic extract. H_2_O_2_ (4 mM; 3%) was added to the medium for 1.5 h. The fluorescence intensity of DCFDA was read after 45 min of incubation. Trolox was used as a positive control, and 0.5% DMSO, in which the metabolites were dissolved, was used as a negative control. The values are the mean’s six values for each experimental point of two independent biological replicates. The statistical analysis was performed with a two-way ANOVA using Tukey’s multiple comparison test. The levels of significance between the points of expression are indicated (**** *p* < 0.001, ** *p* < 0.05, * *p* < 0.01).

The cytotoxicity and antioxidant activity of the crude hydroalcoholic extract and the LLE and SPE fractions obtained from *S. pinnata*’s aerial parts were evaluated as described in Section 4. Crude extract exhibited noticeable antioxidant activity on HeLa and HaCaT cells (Figure 3, B panels) alongside an almost complete absence of toxicity (Figure 3, A panels).

**Figure 4:**
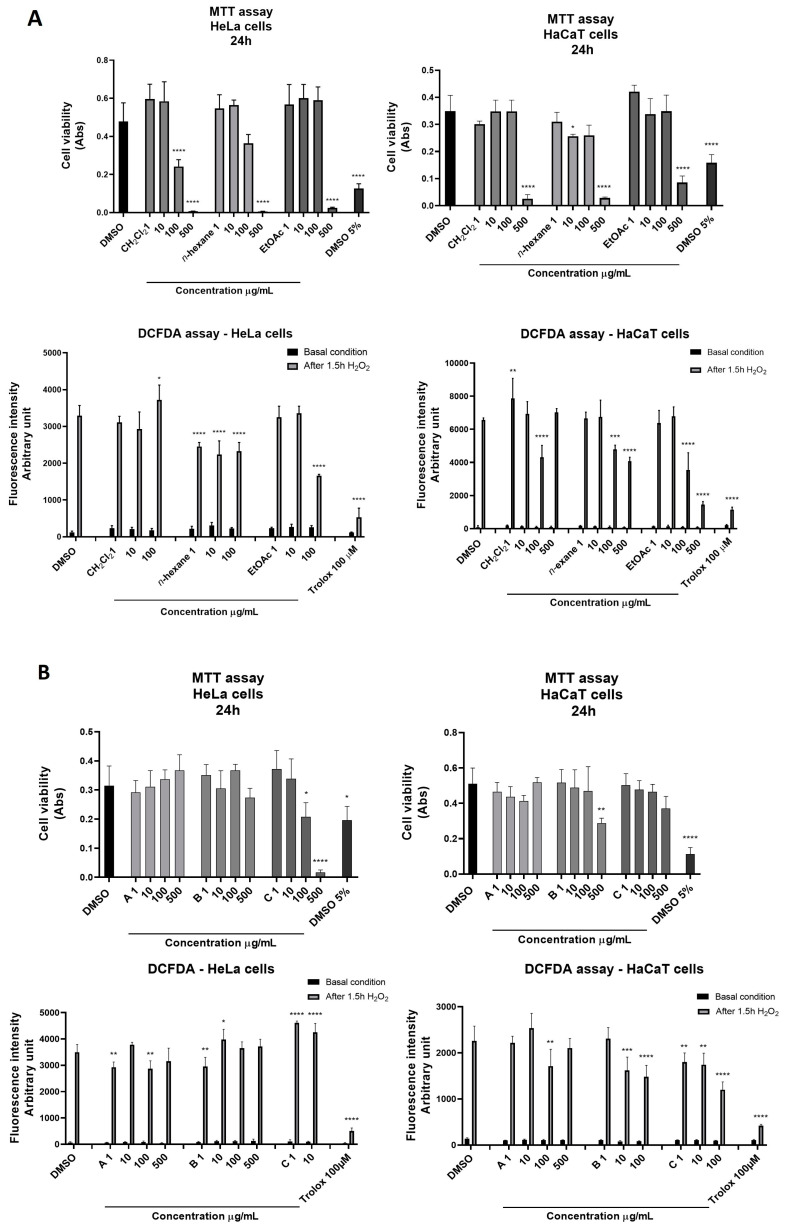
(**A**) MTT assays at 24 h of the CH_2_Cl_2_, *n*-hexane, and EtOAc fractions obtained through the LLE procedure on HeLa (left panel) and HaCaT cells (right panel) at concentrations of 1, 10, 100, and 500 μg/mL. Lycorine or 5% DMSO was used as a positive control. DCFDA assay of the CH_2_Cl_2_, *n*-hexane, and EtOAc fractions on HeLa (left panel) and HaCaT cells (right panel). Trolox was used as a positive control. (**B**) MTT viability test. HeLa and HaCaT cells were incubated with the indicated amount of the A, B, and C fractions obtained through the SPE technique for 24 h. The values were the mean’s six values for each experimental point of two independent biological replicates. Each mean was compared using a Dunnett’s multiple comparisons test of a one-way ANOVA (*p*-value * *p* < 0.01, ** *p* < 0.05, *** *p* < 0.001; **** *p* < 0.0001). (**B**) panels, DCFDA assay. HeLa and HaCaT cells were seeded and pre-treated for 4 h with no toxic concentrations of the A, B, and C fractions obtained through the SPE technique. H_2_O_2_ (4 mM; 3%) was added to the medium for 1.5 h. The fluorescence intensity of DCFDA was read after 45 min of incubation. Trolox was used as a positive control, and DMSO, in which the metabolites were dissolved, was used as a negative control. The values are the mean’s six values for each experimental point of two independent biological replicates. The statistical analysis was performed with a two-way ANOVA using Tukey’s multiple comparison test. The levels of significance between the points of expression are indicated (**** *p* < 0.001, *** *p* < 0.01, ** *p* < 0.05, * *p* < 0.01).

The cytotoxicities of the LLE fractions (*n*-hexane, CH_2_Cl_2_, and EtOAc organic extracts) were evaluated in HeLa and HaCaT cells at the indicated concentrations (Figure 4, A panels).

At 500 μg/mL, all three fractions were highly toxic to both cell lines. Moreover, the CH_2_Cl_2_ fraction caused significant toxicity at a concentration of 100 μg/mL in HeLa cells. (Figure 4, A upper panels). The antioxidant activity of the LLE fractions was evaluated by performing the DCFDA assays at concentrations showing minimal or no toxicity to the cells. As shown in Figure 4A’s lower panels, all three fractions at the concentration of 100 μg/mL caused a 30% reduction of ROS in HaCaT cells, while in the HeLa cells, the *n*-hexane fraction was effective at 1 and 10 μg/mL, causing a 25% and 30% ROS reduction, respectively.

Considering the advantage in yield and sustainability of the SPE method, we focused on the fractions obtained using such a procedure. Therefore, SPE fractions A, B, and C were first tested for cytotoxicity. As shown in Figure 4, fraction A was not toxic, fraction B was toxic to HaCaT cells at 500 μg/mL (50% of the residual activity), while fraction C was toxic to HeLa and HaCaT cells at a concentration of 100 and 500 μg/mL, respectively. Given these results, we evaluated the antioxidant activity of the SPE fractions at concentrations showing minimal or no toxicity to cells by performing DCFDA assays (Figure 4, B panels). In HeLa cells, none of the fractions showed effective antioxidant activity (Figure 4B, left panel). Remarkably, in HaCaT cells, fractions B and C exhibited significant antioxidant activity. Fraction C had the highest antioxidant power (a 50% reduction of ROS at 100 mg/mL), coupled with a substantial absence of toxicity (Figure 4B, right panel).

### 2.3. Purification and Identification of Pure Metabolites from S. pinnata

**Figure 5:**
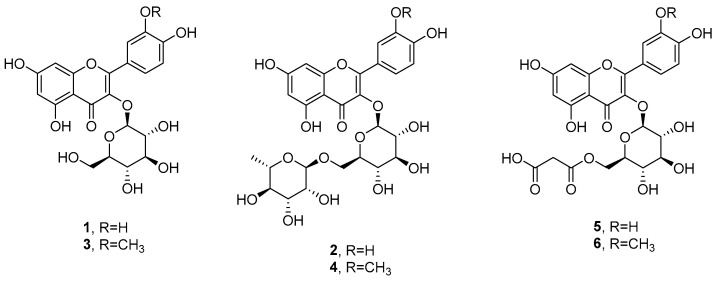
Chemical structures of the compounds isolated from *S. pinnata*: isoquercetin (**1**), rutin (**2**), isorhamnetin glucoside (**3**), narcissoside (**4**), quercetin malonyl glucoside (**5**), and isorhamnetin malonyl glucoside (**6**).

The C fraction from the SPE was chromatographed as detailed in the Section 4 to afford six flavonol glycosides (Figure 5). Briefly, the residue of the SPE C fraction was purified using two steps of TLC on direct and reverse phases, yielding six pure compounds. By comparing their spectroscopic data (essentially the ^1^H NMR, Figures S1–S6) with those reported in the literature, they were identified as isoquercetin (**1**) [14], rutin (**2**) [15], isorhamnetin glucoside (**3**) [14], narcissoside (**4**) [16], quercetin malonyl glucoside (**5**) [17], and isorhamnetin malonyl glucoside (**6**) [17]. Their identification was confirmed using the data obtained from the ESIMS spectra recorded in the negative mode (Figures S7–S12) that showed the deprotonated pseudomolecular ion [M-H]^-^ peaks at *m*/*z* 463, 609, 477, 623, 549, and 563 (**1**–**6**). Finally, their configuration was confirmed by comparing the specific optical rotation data with those reported in the literature [14,15,16,17].

### 2.4. Cytotoxicity and Antioxidant Properties of the Pure Metabolites from S. pinnata

**Figure 6:**
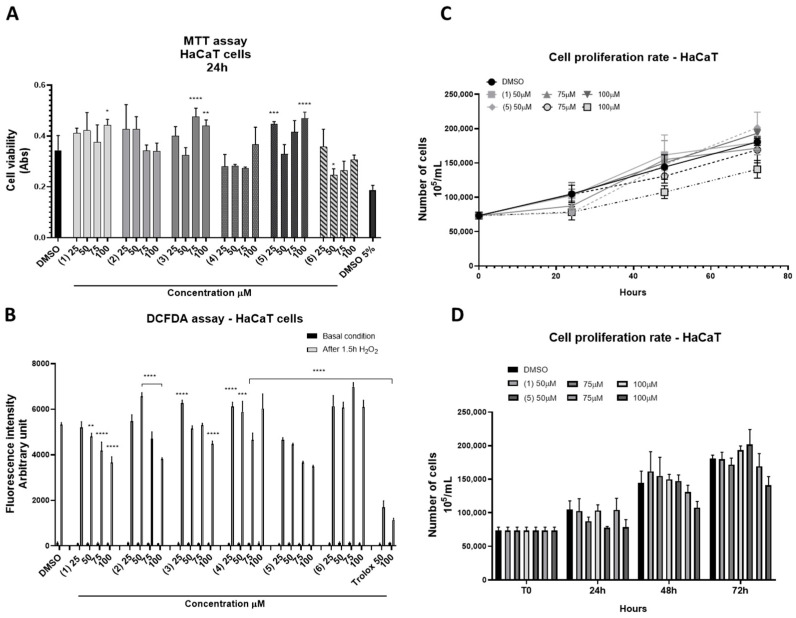
(**A**) MTT viability test. HaCaT cells were incubated with the indicated amount of pure metabolites (**1**–**6**) obtained from fraction C of *S. pinnata* for 24 h. The values were the mean’s six values for each experimental point of two independent biological replicates. Each mean was compared using a Dunnett’s multiple comparisons test of a one-way ANOVA (*p*-value * *p* < 0.01, ** *p* < 0.05, *** *p* < 0.001; **** *p* < 0.0001). (**B**) DCFDA assay. HaCaT cells were seeded and pre-treated for 4 h with no toxic concentrations of the pure metabolites (**1**–**6**) obtained from fraction C of *S. pinnata*. H_2_O_2_ (4 mM; 3%) was added to the medium for 1.5 h. The fluorescence intensity of DCFDA was read after 45 min of incubation. Trolox was used as a positive control, and DMSO, in which the metabolites were dissolved, was used as a negative control. The values are the mean’s six values for each experimental point of two independent biological replicates. The statistical analysis was performed with a two-way ANOVA using Tukey’s multiple comparison test. The levels of significance between the points of expression are indicated (**** *p* < 0.001, *** *p* < 0.01, ** *p* < 0.05). (**C**,**D**) Cell growth profile. HaCaT cells were seeded and treated with isoquercitin (**1**) and quercetin malonyl glucoside (**5**) at the indicated concentrations. The cells were counted with Scepter at T0, 24, 48, and 72 h of treatments, and the number was compared to the untreated cells. The results are the mean ± SEM of three independent biological experiments relative to the experimental control (DMSO). The statistical analysis was performed with a one-way ANOVA using Dunnett’s multiple comparison test.

HaCaT cells are widely used as a model for studying the effect of natural compounds on keratinocyte proliferation and differentiation. Therefore, pure metabolites (**1**–**6**) obtained from fraction C of *S. pinnata* were tested for their effect on cell viability in HaCaT cells using the MTT assay. Each metabolite was tested at the range of concentration between 25 and 100 μM. As shown in Figure 6, A panel, the HaCaT cell viability was stimulated using treatments with the metabolites isoquercetin (**1**), rutin (**2**), isorhamnetin glucoside (**3**), and quercetin malonyl glucoside (**5**), although to a different extent.

No significant effect on cell viability was observed by treating the cells with the metabolite narcissoide (**4**), while a 40% reduction in cell viability was observed by treating the cells with isoramnetin malonil glucoside (**6**) at the concentration of 50 mg/mL. The antioxidant activity of the pure metabolites (**1**–**6**) was evaluated using a DCFDA assay in the same range of concentration. As shown in Figure 6, B lower panel, among all the metabolites assayed, isoquercetin (**1**) and quercetin malonyl glucoside (**5**) exhibited dose-dependent antioxidant power. Moreover, neither isoquercetin nor malonyl glucoside stimulated the rate of cell proliferation at the tested concentrations (Figure 6C,D).

### 2.5. In Vitro Wound Healing Assay

**Figure 7:**
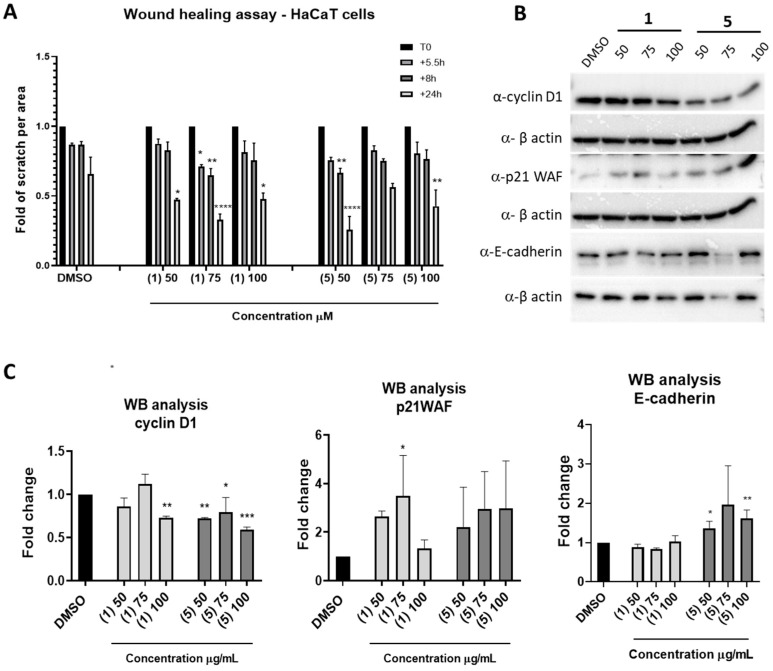
Wound healing assay. (**A**) The HaCaT cell monolayer was scratched in the center with a sterile tip and treated with isoquercetin (**1**) or quercetin malonyl glucoside (**5**) at 50 μM, 75 μM, and 100 μM. The statistical analysis was performed with a two-way ANOVA using Dunnett’s multiple comparison test (*p*-value * *p* < 0.01, ** *p* < 0.05, **** *p* < 0.0001). The results are the mean ± SEM of three independent biological experiments relative to the experimental control (DMSO). (**B**) Representative image of the Western blot analysis of the extracts from HaCaT keratinocytes treated for 24 h with 50, 75, and 100 μg/mL of (**1**) and (**5**). DMSO (cells in 0.1% DMSO, negative control). The immunoblots were probed with p21WAF, cyclin D1, and E-cadherin antibodies. β-actin was used as a loading control. (**C**) The protein bands were quantified using ImageLab software version 4.1 (Bio-Rad, Hercules, CA, USA). The statistical analyses were carried out using an ordinary one-way ANOVA followed by Dunnett’s multiple comparison test (*** *p* < 0.001, ** *p* < 0.01, * *p* < 0.05) and Student’s *t*-test (** *p* < 0.01, * *p* < 0.05) for E-cadherin analysis. The results are the mean ± SEM of three independent biological experiments relative to the experimental control (DMSO).

The in vitro wound healing activity of isoquercetin (**1**) and quercetin malonyl glucoside (**5**) was determined using a scratch assay, which is a model of a wound in which monolayer keratinocytes react to the disruption of contact between cells and are stimulated to proliferate and migrate, thus repairing the wound [18]. For this purpose, HaCaT cells were seeded in a 12-well plate and allowed to grow to 90% confluence. To mimic a wound, a linear scratch was created in the center of a confluent cell monolayer with a sterile 20 μL tip. The cells were washed and then incubated with fresh control medium or medium supplemented with isoquercetin (**1**) or quercetin malonyl glucoside (**5**) at 50 μM, 75 μM, and 100 μM to judge the rate of cell migration [19]. As shown in Figure 7A, both metabolites showed healing-promoting activity. In particular, quercetin malonyl glucoside (**5**) was the most effective by reducing the wound up to 25% after 24 h of incubation at the concentration of 50 mM. Interestingly, in HaCat keratinocytes treated with malonyl glucoside, we observed an increase in E-cadherin and p21WAF and a reduction in cyclin D1 protein expression (Figure 7B,C).

### 2.6. Antimicrobial and Microbicidal Assays

The survival and die-off rates of *Staphylococcus aureus* ATCC^®^6538 (Table 1), *Pseudomonas aeruginosa* ATCC^®^9027 (Table 2), and *Candida albicans* ATCC^®^14053 (Table 3) were evaluated following contact with fraction C at 150 µg/mL and 100 µg/mL concentrations and isoquercetin (**1**) and quercetin malonyl glucoside (**5**) at 100 µM. The activity of fraction C (150 µg/mL) against *Staphylococcus aureus* ATCC^®^6538 evidenced a 96.97% die-off rate of the microbial load after 30 min of contact time. Fraction C (100 µg/mL) showed a 90.93% die-off rate. The activities of isoquercetin (**1**) and quercetin malonyl glucoside (**5**) (at a 100µM concentration) demonstrated low die-off rates against *S. aureus* ATCC^®^6538, ranging from 8 to 10% (Table 1).

Assays conducted to assess the effects of 150 µg/mL and 100 µg/mL of fraction C on *Pseudomonas aeruginosa* ATCC^®^9027 brought a reduction of 39.64% and 5.60% loads, respectively. Based on the data, the microbial loads of *P. aeruginosa* ATCC^®^9027 sensibly reduced following contact with isoquercetin (**1**) (85.77%) and quercetin malonyl glucoside (**5**) (72.88%) (Table 2).

The antifungal evaluation targeting *Candida albicans* ATCC^®^14053 highlighted a statistically significant (*p*-values < 0.05 and *p* ≤ 0.0001, respectively) die-off efficacy of fraction C on the yeast, ranging from 21.02% (for the 150 µg/mL tested concentration) to 52.68% (for the 150 µg/mL tested concentration) (Table 3).

The outcomes of the tests conducted with 100 µM isoquercetin (**1**) and quercetin malonyl glucoside (**5**) did not show statistically significant fungal load reductions. Thus, considering the obtained results and taking into account the assayed concentrations, it is possible to affirm that fraction C demonstrated a high die-off efficacy (a reduction from 91% to 97%) towards *S. aureus* ATCC^®^6538 and a milder effect on *C. albicans* ATCC^®^14053, while isoquercetin (**1**) and quercetin malonyl glucoside (**5**) showed a sensible bactericidal effect (a reduction from 73% to 86%) on *P. aeruginosa* ATCC^®^9027.

## 3. Discussion

The data from the literature indicate that *Staphylea* spp. extracts contain bioactive metabolites with potential applications in the cosmeceutical industry [6].

From the aerial parts of the hydroalcoholic extract of *Staphylea pinnata* L. collected in Italy, we isolated six pure compounds (compounds **1**–**6**) using SPE and chromatographic techniques. They were identified using spectroscopic, spectrometric, and optical methods. Compounds **1**, **2**, and **5** are quercetin glycosides, while compounds **3**, **4**, and **6** are isorhamnetin glycosides belonging to the flavonol group of flavonoids, a class of polyphenolic secondary metabolites found in plants [20]. These plant-specialized metabolites have important functions in plant growth and development [21] and show several biological activities [22,23].

The six pure metabolites were tested for their biological activity on human tumor-derived HeLa cells and immortalized HaCaT keratinocytes. In human immortalized keratinocytes, fractions B and C obtained using SPE showed remarkable antioxidant activities. Such activities were not observed in tumor-derived HeLa cells. The variation in the antioxidant properties of plant extracts or their fractions across different cell types is not surprising and can be attributed to several factors. For instance, different cells have varying levels of oxidative stress and antioxidant defense mechanisms. Cells with different metabolic activities, receptors, activated signal pathways, and redox statuses may process and utilize antioxidants differently. This can affect the efficacy of plant extracts or partially purified fractions in exhibiting antioxidant properties. In particular, cancer-derived cells are usually characterized by constitutively activated antioxidant responses that promote their survival and can obscure the activity of antioxidant metabolites [24]. In HaCaT keratinocytes, fraction C turned out to be the most interesting as it had the highest antioxidant power coupled with a substantial absence of toxicity. Among the metabolites isolated from SPE fraction C, none of them were toxic to keratinocytes. Moreover, both isoquercetin (**1**) and quercetin malonyl glucoside (**5**) exhibited a significant capacity to reduce ROS levels induced by hydrogen peroxide, thus suggesting that quercetin glycosides, which are abundant in *S. pinnata*, show potential for being effective ingredients in cosmeceutical products designed to protect the skin from oxidative stress. The wound healing process was improved in keratinocytes treated with isoquercetin and quercetin malonyl glucoside, although our data indicated that quercetin malonyl glucoside was the most effective in wound repair. Wound repair requires keratinocyte proliferation and migration. However, the lack of enhanced cell proliferation in HaCaT treated with quercetin malonyl glucoside suggests that the improvement of the wound healing process may reflect an increase in cell migration rather than proliferation. This appears to be confirmed by the observed reduction in cyclin D1 and the increase in the p21WAF cell cycle inhibitor. On the other hand, the treatment of HaCaT with quercetin malonyl glucoside enhanced the expression level of E-cadherin, which facilitates the formation of adherent junctions between adjacent epithelial cells, thus promoting efficient wound closure [25].

The available bibliography highlighted sensitive antimicrobial and antifungal activities towards several bacteria and yeasts of either quercetin-derived substances or quercetin and malonyl glucoside-containing plant extracts. A study published in 2020 reported the potential synergistic activity of quercetin with antibiotics against multidrug-resistant clinical strains of *Pseudomonas aeruginosa*. Quercetin was indeed described as capable of targeting quorum sensing and demonstrated to be particularly active against biofilm-forming microorganisms. The research highlighted an ≥ 80% inhibition of *Pseudomonas aeruginosa* strains treated with blends of quercetin and selected antibiotic combinations [26]. Furthermore, quercetin exhibited antifungal activity against *Candida albicans* by means of the induction of yeast apoptosis through mitochondrial dysfunction following the accumulation of Mg^2+^ [27]. Tan et al. (2023) [28] confirmed the results of the present study, discovering that the minimum inhibitory concentration (MIC) and minimal fungicidal concentration (MFC) of quercetin for *C. albicans* were >128 μM and >512 μM, respectively [28]: a quercetin concentration lower than 128 μM, such as 100 μM, was not capable of inhibiting *C. albicans* [28]. With regards to quercetin malonyl glucoside, Abdalla et al. (2022) [29] reported the extraction of a substance from red lettuce: the extract showed antibacterial activity against *Pseudomonas aeruginosa* [29]. Quercetin malonyl glucoside was additionally described as holding an effective antibacterial activity towards *Staphylococcus aureus*, *S. epidermidis*, *S. faecalis*, and *S. pyogenes* [30]. Sensitive antibacterial activity was additionally described by Mugo and Njenga (2020) [31] for essential oil from an *Ixora scheffleri* subspecies, *keniensis*, from which the MS spectrum also detected the presence of malonyl glucoside quercetin: the extracts showed antimicrobial properties against human pathogen strains, such as *S. aureus*, *B. subtilis*, *E. coli*, *P. aeruginosa*, and *C. albicans* [31].

## 4. Materials and Methods

### 4.1. Plant Material

The aerial parts of *S. pinnata* were collected in late August 2023 in a thermophilic mixed wood at an elevation of 600 m in the proximity of Luzzano, a village belonging to the municipality of Moiano (Benevento, Italy). The identification of the plant material was performed according to the Flora of Italy, comparing the collected material to reference vouchers (PI 010576, https://erbario.unipi.it/it/erbario/view?id=1283285, accessed on 18 May 2024). The collection was carried out in a period far from flowering and fruiting, collecting the terminal part of the leafy twigs from about 30 individuals, spaced 5–10 m apart to reduce the risk of sampling clonal individuals. Distilled water was used to rinse the plant material and remove the dust particles. The plant was then dried for a few days in the air at room temperature and ground in a blender.

### 4.2. General Experimental Procedures

The solid phase extraction (SPE) was performed using SUPELCO Supelclean LC-18 SPE 10 g/60 mL cartridges (Merck, Darmstadt, Germany) on a SUPELCO Visiprep™ SPE Vacuum Manifold system (Merck, Darmstadt, Germany). Analytical and preparative thin-layer chromatography (TLC) was performed on silica gel plates (Kieselgel 60, F_254_, 0.25 and 0.5 mm, respectively) or reverse phase (Whatman, KC18, F_254_, 0.20 mm) (Merck, Darmstadt, Germany) plates, and the compounds were visualized via exposure to UV light and/or iodine vapors and/or by spraying first with 10% H_2_SO_4_ in MeOH, and then with 5% phosphomolybdic acid in EtOH, followed by heating at 110 °C for 10 min. All the solvents employed were supplied by Sigma-Aldrich (Milan, Italy). ^1^H nuclear magnetic resonance (NMR) spectra were recorded at 400 or 500 MHz on Bruker (Karlsruhe, Germany) or Varian (Palo Alto, CA, USA) spectrometers, respectively. Electrospray ionization (ESI) mass spectra and liquid chromatography (LC/MS) analyses were performed using the LC/MS TOF system AGILENT 6230B, HPLC 1260 Infinity (Milan, Italy) in the negative modality. Optical rotations were measured on a Jasco (Tokyo, Japan) P-1010 digital polarimeter. The purity of the purified compounds was >98%, as ascertained via ^1^H NMR and HPLC analyses.

### 4.3. Extraction and Purification of the Metabolites

A total of 10 g of dried aerial parts of *S. pinnata* were minced with a blender and macerated under stirred conditions in a solution of methanol (MeOH)/H_2_O 1:1, *v*/*v* (300 mL) for 3 days at room temperature. The resulting suspension was filtered, resulting in a hydroalcoholic crude extract. Two aliquots of this latter extract were further fractionated by performing a liquid–liquid extraction (LLE) and a solid-phase extraction (SPE).

The first aliquot (100 mL) of the hydroalcoholic extract was subjected to stepwise extractions with *n*-hexane (3 × 100 mL), dichloromethane (CH_2_Cl_2_) (3 × 100 mL), and after removing MeOH under reduced pressure, with ethyl acetate (EtOAc) (3 × 100 mL). Each extract was dried over anhydrous Na_2_SO_4_ and filtered, and the residual solvent was evaporated under reduced pressure, yielding 10.35 mg (*n*-hexane), 15.90 mg (CH_2_Cl_2_), and 36.87 mg (EtOAc) of organic extracts.

The second aliquot (100 mL) of the hydroalcoholic extract was lyophilized after removing MeOH under reduced pressure. The extract obtained was suspended in 60 mL of distilled water and loaded onto an SPE column, which was previously conditioned with 60 mL of MeOH and equilibrated with 120 mL of distilled water. The purification yielded five fractions (A, B, C, D, and E) obtained via a stepwise elution with H_2_O (60 mL), MeOH/H_2_O 3:7, *v*/*v* (120 mL), MeOH/H_2_O 7:3, *v*/*v* (120 mL), acetonitrile (CH_3_CN) (120 mL), and CH_2_Cl_2_/MeOH 9:1, *v*/*v* (60 mL), respectively. Successively, the organic solvents were removed under reduced pressure, and the eventual residual water phases were lyophilized, yielding 570.59 mg (A), 310.94 mg (B), 132.01 mg (C), 4.36 mg (D), and 3.27 mg (E), respectively.

The extracts and fractions obtained by applying the two procedures (LLE and SPE) were tested for biological activities, as reported below. Furthermore, the residue of SPE fraction C was purified using two subsequent steps of preparative TLC eluted with EtOAc/MeOH/H_2_O (80:12:8, *v*/*v*/*v*) and TLC in reverse phase by eluting it with MeOH/H_2_O (6:4, *v*/*v*), affording isoquercetin (**1**, 3.78 mg), rutin (**2**, 7.50 mg), isorhamnetin glucoside (**3**, 4.78 mg), narcissoside (**4**, 2.69 mg), quercetin malonyl glucoside (**5**, 5.81 mg), and isorhamnetin malonyl glucoside (**6**, 3.62 mg) as amorphous solids. The extraction and purification process was repeated three times to accumulate the pure compounds for chemical and biological characterization.

Isoquercetin (**1**): or quercetin 3-*O*-β-D-glucopyranoside, amorphous solid, [α]^25^_D_ −12.6 (*c* 0.1, MeOH) (ref. [14] [α]^25^_D_ −10.7 (*c* 0.4, MeOH)); ^1^H NMR data were in agreement with those previously reported by He et al. 2017 [14]; ESI MS (-): *m*/*z* 463 [M−H]^−^.

Rutin (**2**): or quercetin 3-*O*-β-D-rutinoside, amorphous solid, [α]^25^_D_ −14.9 (*c* 0.1, MeOH) (ref. [32] [α]^25^_D_ −16.6 (*c* 0.1, MeOH)); ^1^H NMR data were in agreement with those previously reported by Kazuma et al. 2003 [15]; ESI MS (-): *m*/*z* 609 [M-H]^−^.

Isorhamnetin glucoside (**3**): or isorhamnetin-3-*O*-β-D-glucopyranoside, amorphous solid, [α]^25^_D_ −9.6 (*c* 0.1, MeOH) (ref. [14] [α]^25^_D_ −7.8 (*c* 0.2, MeOH)); ^1^H NMR data were in agreement with those previously reported by He et al. 2017 [14]; ESI MS (-): *m*/*z* 477 [M-H]^−^.

Narcissoside (**4**): or isorhamnetin-3-*O*-β-D-rutinoside, amorphous solid, [α]^25^_D_ −36.6 (*c* 0.1, MeOH) (ref. [33] [α]^20^_D_ −38.7 (*c* 0.3, MeOH)); ^1^H NMR data were in agreement with those previously reported by Eskalieva et al. 2004 [16]; ESI MS (-): *m*/*z* 623 [M-H]^−^.

Quercetin malonyl glucoside (**5**): or quercetin-3-*O*-(6″-*O*-malonyl)-β-D-glucopyranoside, amorphous solid, [α]^25^_D_ −14.6 (*c* 0.1, MeOH) (ref. [34] [α]^25^_D_ −15.7 (*c* 0.1, MeOH)); ^1^H NMR data were in agreement with those previously reported by Wald et al. 1989 [17]; ESI MS (-): *m*/*z* 549 [M-H]^−^.

Isorhamnetin malonyl glucoside (**6**): or isorhamnetin-3-*O*-(6″-*O*-malonyl)-β-D-glucopyranoside, amorphous solid, [α]^25^_D_ −8.2 (*c* 0.1, MeOH) (ref. [35] [α]^25^_D_ −8.7 (*c* 0.1, MeOH)); ^1^H NMR data were in agreement with those previously reported by Wald et al. 1989 [17]; ESI MS (-): *m*/*z* 563 [M-H]^−^.

### 4.4. Cell Culture and Reagents

HeLa cells were obtained from the American Type Culture Collection (ATCC CCL-2) and cultured in DMEM supplemented with 10% FBS. HaCaT, spontaneously immortalized keratinocytes from adult skin, were purchased from Service Cell Line (GmBH, Eppelheim, CLS, Germany). The cells (10–14 passages) were cultured in Dulbecco’s Modified Eagle’s Medium (DMEM, Sigma Chemical Co, St. Louis, MO, USA) supplemented with 10% fetal bovine serum (FBS, Hyclone Laboratories, Inc. Logan, UT, USA) at 37 °C in a humified atmosphere of 5% CO_2_ and routinely tested for mycoplasma contamination.

### 4.5. Western Blot Analysis

Whole-cell extracts (20 μg) were separated using sodium dodecyl sulfate-polyacrylamide gel electrophoresis (SDS-PAGE), subjected to a Western blot, and incubated overnight at 4 °C with antibodies against p21WAF, cyclin D1 from Cell Signaling Technologies (Boston, MA, USA), and e-cadherin and β-actin from Santa Cruz Biotechnology (Dallas, TX, USA). Each experiment was run in triplicates. The signal intensities of the bands were quantified using Quantity One analysis software (Version Number 2, Biorad Laboratories, London, UK) and analyzed using GraphPad Prism 8.0.2 software (GraphPad, San Diego, CA, USA).

### 4.6. DCFDA Assay

The antioxidant activities of the total hydroalcoholic extracts, fractions, and pure metabolites from *S. pinnata* L. leaves were measured using 2′−7′dichlorofluorescein diacetate (DCFDA), a non-fluorescent compound permeable to the cell membrane, which can be rapidly oxidized by reactive oxygen species (ROS), producing a fluorescent compound, as previously described [36]. In brief, 2 × 10^4^ cells were treated with increasing doses of extract, fractions, or purified metabolites as indicated. The medium was removed after 4 h, and 4 mM (3%) H_2_O_2_ was added for 1.5 h. The cells were washed with PBS, and a fresh medium with DCFDA (30 mM) was added for 45 min; then, DCFDA was removed by washing in 1X PBS, and the cells were harvested. The measurement of the ROS was obtained using the Sinergy H4 microplate reader Gen5 2.07 (Thermofisher, Waltham, MA, USA). The fluorescence emitted from the cells treated with DCFDA was compared to the untreated cells. Trolox was used as a positive control. The values shown in the plot are the mean ± SD of sixfold determinations. The means and the standard deviations were calculated as biological triplicates using GraphPad Prism 8.0.2 software (GraphPad, San Diego, CA, USA).

### 4.7. Cell Viability Assay

The cell viability was evaluated by measuring the reduction in 3-(4,5-dimethylthiazol-2) 2,5-diphenyltetrazolium bromide (MTT) to formazan by the mitochondrial enzyme succinate dehydrogenase [37]. Briefly, a day before, 1 × 10^4^ cells were seeded on 96-well plates and treated with increasing concentrations of total extract or metabolites for 24 h. MTT (0.5 mg/mL) in PBS was then added to the wells and incubated for 3 h at 37 °C in a humidified atmosphere. The reaction was stopped by the removal of the supernatant followed by dissolving the formazan product in acidic isopropanol, and the optical density was measured with a Sinergy H4 microplate reader Gen5 2.07 (Thermofisher, Waltham, MA, USA) using a 570 nm filter. Under these experimental conditions, no undissolved formazan crystals were observed. The cell viability was assessed by comparing the optical density of the treated samples compared to the controls.

### 4.8. Cell Growth Profile

A total of 3.1 × 10^3^ HaCaT cells were seeded in a 12-well plate; the cells were serum-starved for 24 h; after starvation, isoquercetin (**1**) and quercetin malonyl glucoside (**5**) were added at different concentrations. Every 24 h, the cells were gently rinsed with 1× PBS, trypsinized, and counted. The count was confirmed via Scepter 2.0 analysis (Millipore, Burlington, MI, USA), as previously described [38].

### 4.9. Wound Healing Assay

A wound healing assay was performed by seeding 9 × 10^4^ HaCaT cells in a 12-well plate. We allowed them to grow to 90% confluence. To mimic a wound, a scratch was created in the center of a confluent cell monolayer with a sterile 20 μL tip. After scratching, the cells were washed twice with 1X PBS, and fresh control medium or medium supplemented with isoquercetin (**1**) or quercetin malonyl glucoside (**5**) at 50 μM, 75 μM, and 100 μM was added.

Images were obtained immediately after the scratch (t0) at 5.5, 8, and 24 h after treatment using a LEICA microscope. The wound area was analyzed using ImageJ software (Version 1.54i) by comparing the residual area of the treated samples to the control.

### 4.10. Antimicrobial Assays

The bactericidal/antibacterial activities of SPE fraction C and the pure compounds isoquercetin (**1**) and quercetin malonyl glucoside (**5**) were evaluated on two target microorganisms: *Staphylococcus aureus* ATCC^®^6538 and *Pseudomonas aeruginosa* ATCC^®^9027. The fungicidal/antifungal activity was assessed towards *Candida albicans* ATCC^®^14053. Single cultures of the three strains were grown according to the microorganism requirements: *S. aureus* ATCC^®^6538 for 24–48 h at 37 °C (UNI EN ISO 6888-1:2021) [39], *P. aeruginosa* ATCC^®^9027 for 24 h at 37 ± 1 °C (UNI EN ISO 13720:2010) [40], and *C. albicans* ATCC^®^14053 for 48 h at 22 ± 1 °C (UNI EN ISO 21527-1:2008) [41]. Microorganism inocula were kept in constant shaking conditions at 200 rpm in 30 mL of Tryptic Soy Broth (TSB, Thermo Fisher Scientific, USA) for the bacteria and 30 mL of Sabouraud Dextrose Broth (SAB, Thermo Fisher Scientific, USA) for the yeast in 50 mL total-volume sterile tubes. To obtain an equal 10^8^ cell/mL concentration of both microorganisms, a spectrophotometer (Hach Lange DR6000, Hach, USA) was used to measure the absorbance at 560 nm (bacteria) and 600 nm (yeast): an optical density (OD) of 0.125 was used as an indicator of 10^8^ cell/mL concentration. The inocula were serially diluted to reach the target concentration of 10^7^ CFU/mL in 10 mL. Fraction C’s activity was tested at two concentrations: 150 µg/mL and 100 µg/mL. Assays with isoquercetin (**1**) and quercetin malonyl glucoside (**5**) were performed by selecting a 100 µM concentration, resulting in the lowest concentration that did not show toxicity to the cells.

The experimental protocol was set up according to UNI EN 1276:2020 [42], employing the time-kill test [43] for determining the bactericidal or fungicidal effect of the samples under analysis, with a 30-minute time of contact. The selected concentrations of the three samples, diluted in 50 µL of dimethyl sulfoxide (DMSO, Sigma-Aldrich, USA), were added to 6-well plates aimed at testing 0.9% NaCl suspensions of 10 mL of bacteria or yeast at a microbial load of 10^6^ CFU/mL, maintained in constant shaking and at the optimal temperature (37 ± 1 °C for the bacteria, 22 ± 1 °C for the yeast) for a 30 min contact time. Negative controls were prepared with the sole bacterial or yeast inocula with 50 µL of DMSO without the addition of the test substances. The addition of the bacteria suspension of 100 µg/mL chloramphenicol and the yeast inoculum of 100 µg/mL econazole served as the positive controls. Shortly after the inoculation (t_0min_) of the test substances and after 30 min (t_30min_), aliquots were sampled, the activities were determined, verifying the numbers of surviving bacteria in each sample, and the reduction was calculated. The microbiological analyses were conducted following the ISO guidelines specific to each indicated microorganism, employing selective and specific agarized culture media for *S. aureus* (Baird Parker Agar Base, Thermo Fisher Scientific, USA, ISO 6888-1:2021), *P. aeruginosa* (Pseudomonas Agar Base, Thermo Fisher Scientific, USA, UNI EN ISO 13720:2010), and *C. albicans* (Dichloran Rose Bengal Chloramphenicol Agar Base, Thermo Fisher Scientific, USA, UNI EN ISO 21527-1:2008). The samples were analyzed in independent triplicates to validate the results. The reported results refer to the mean values of the three tests executed for each microorganism.

### 4.11. Statistical Analysis

The statistical analyses were carried out using GraphPad Prism version 8.1.2 (https://www.graphpad.com/scientific-software/prism/, accessed on 19 May 2024). The data were represented as the mean and standard deviation, and were analyzed for statistical significance using an ordinary one-way analysis of variance (ANOVA) and multiple comparisons and Student’s *t*-test. For all tests, *p* < 0.05 was considered to indicate a statistically significant difference.

For the antimicrobial assays, the data were presented as the three replicates of the mean ± standard error (SE). XLSTAT 2014.5.03 (Addinsoft 1995–2014, France) was used for the statistical analysis: a one-way ANOVA was performed with post-test Tukey correction, and the comparisons of the samples with the negative controls were conducted by selecting the two-sided Dunnett’s test: a *p*-value < 0.05 was considered statistically significant, and in the graphical plots, was denoted as * *p* ≤ 0.05 and ** *p* ≤ 0.0001.

## 5. Conclusions

Our study demonstrated that among the flavonol glycosides isolated from the hydroalcoholic extract of the aerial parts of *Staphylea pinnata* L., isoquercetin and quercetin malonyl glucoside had powerful antioxidant, antimicrobial, and wound healing-promoting activities in human keratinocytes. Therefore, they are of clinical relevance in skin repair and are promising antiaging chemicals for the cosmeceutical industry.

## Figures and Tables

**Table 1:** Antibacterial/bactericidal susceptibility tests conducted on *Staphylococcus aureus* ATCC^®^6538 [10^6^ CFU/mL] ^1^.

Sample	Die-Off Concentration (Mean ± SE) [CFU/mL]	Log CFU Reduction	Die-Off Rate [%]	*p*-Value
Negative control [50 µL DMSO]	1,750,000 ± 50	2 × 10^5^	7.89	-
Positive control [100 µg/mL chloramphenicol]	5500 ± 15	2 × 10^6^	99.71	≤0.0001
Fraction C [150 µg/mL]	57,525 ± 4	2 × 10^6^	96.97	≤0.0001 **
Fraction C [100 µg/mL]	172,267 ± 15	2 × 10^6^	90.93	≤0.0001 **
Isoquercetin (**1**) [100 µM]	1,708,000 ± 125	2 × 10^5^	10.11	>0.05 *
Quercetin malonyl glucoside (**5**) [100 µM]	1,742,333 ± 89	2 × 10^5^	8.30	>0.05 *

^1^ Die-off concentration (3 replicates mean ± SE, expressed in CFU/mL = colony forming units/mL), log CFU reduction and die-off rate (percentage) of *S. aureus* ATCC^®^6538 following contact with fraction C at two different concentrations: isoquercetin (**1**) and quercetin malonyl glucoside (**5**) at 100 µM. Negative control = 50 µL of DMSO; positive control = 100 µg/mL chloramphenicol; * *p* < 0.05, ** *p* ≤ 0.0001 compared to the negative control (a one-way ANOVA followed by Turkey’s and Dunnett’s post hoc tests).

**Table 2:** Antibacterial/bactericidal susceptibility tests conducted on *Pseudomonas aeruginosa* ATCC^®^9027 [10^6^ CFU/mL] ^1^.

Sample	Die-Off Concentration (Mean ± SE) [CFU/mL]	Log CFU Reduction	Die-Off Rate [%]	*p*-Value
Negative control [50 µL DMSO]	1,636,667 ± 79	4 × 10^4^	2.58	-
Positive control [100 µg/mL ehloramphenicol]	5083 ± 13	2 × 10^6^	99.70	≤0.0001
Fraction C [150 µg/mL]	1,013,283 ± 101	7 × 10^5^	39.69	≤0.0001 **
Fraction C [100 µg/mL]	1,586,000 ± 225	9 × 10^4^	5.60	>0.05 *
Isoquercetin (**1**) [100 µM]	239,000 ± 107	1 × 10^6^	85.77	≤0.0001 **
Quercetin malonyl glucoside (**5**) [100 µM]	455,700 ± 68	1 × 10^6^	72.88	≤0.0001 **

^1^ Die-off concentration (3 replicates mean ± SE, expressed in CFU/mL = colony forming units/mL), log CFU reduction and die-off rate (percentage) of *P. aeruginosa* ATCC^®^9027 following contact with fraction C at two different concentrations: isoquercetin (**1**) and quercetin malonyl glucoside (**5**) at 100 µM. Negative control = 50 µL of DMSO; positive control = 100 µg/mL chloramphenicol; * *p* < 0.05 ** *p* ≤ 0.0001 compared to the negative control (a one-way ANOVA followed by Turkey’s and Dunnett’s post hoc tests).

**Table 3:** Antifungal/fungicidal susceptibility tests conducted on *Candida albicans* ATCC^®^14053 [10^6^ CFU/mL] ^1^.

Sample	Die-Off Concentration (Mean ± SE) [CFU/mL]	Log CFU Reduction	Die-Off Rate [%]	*p*-Value
Negative control [50 µL DMSO]	1,400,000 ± 71	8 × 10^4^	5.41	-
Positive control [100 µg/mL econazole]	46,913 ± 9	1 × 10^6^	96.83	≤0.0001
Fraction C [150 µg/mL]	700,333 ± 72	8 × 10^5^	52.68	≤0.0001 **
Fraction C [100 µg/mL]	1,168,867 ± 220	3 × 10^5^	21.02	<0.05 *
Isoquercetin (**1**) [100 µM]	1,391,500 ± 122	9 × 10^4^	5.98	>0.05 *
Quercetin malonyl glucoside (**5**) [100 µM]	1,359,667 ± 73	1 × 10^5^	8.13	>0.05 *

^1^ Die-off concentration (3 replicates mean ± SE, expressed in CFU/mL = colony forming units/mL), log CFU reduction and die-off rate (percentage) of *C. albicans* ATCC^®^14053 following contact with fraction C at two different concentrations: isoquercetin (**1**) and quercetin malonyl glucoside (**5**) at 100 µM. Negative control = 50 µL DMSO; positive control = 100 µg/mL chloramphenicol; * *p* < 0.05 ** *p* ≤ 0.0001 compared to the negative control (a one-way ANOVA followed by Turkey’s and Dunnett’s post hoc tests).

## Data Availability

The data that support the findings of this study are available from the corresponding author upon reasonable request.

## Supplementary Materials

The following supporting information can be downloaded at: https://www.mdpi.com/article/10.3390/ijms25115582/s1.
