# Supplementary material for: Health-Promoting Properties of Natural Flavonol Glycosides Isolated from Staphylea pinnata L
Source: Int J Mol Sci. 2024 May 21;25(11):5582. doi: 10.3390/ijms25115582 (PMC11171919; doi:10.3390/ijms25115582)
Supplement: Supplementary file 1 [file ijms-25-05582-s001.zip › ijms-2988552-supplementary.pdf]

SUPPORTING INFORMATION

Health-promoting properties of natural flavonol glycosides isolated from *Staphylea pinnata* L.

Ida Paolillo [1], Giuseppina Roscigno [1], Michele Innangi [2], Jesús G. Zorrilla [3,4], Gianmarco Petraglia [3], Maria Teresa Russo [3], Federica Carraturo [1], Marco Guida [1], Alessandra Pollice [1], Alessio Cimmino [3], Marco Masi [3,*] and Viola Calabrò [1]

1 Department of Biology, University of Naples Federico II, Complesso Universitario Monte Sant'Angelo, Via Cintia 4, 80126 Naples, Italy; ida.paolillo@unina.it (I.P.); giuseppina.roscigno@unina.it (G.R.); federica.carraturo@unina.it (F.C.); marco.guida@unina.it (M.G.); apollice@unina.it (A.P.); vcalabro@unina.it (V.C.)

2 EnviXLab, Department of Biosciences and Territory, University of Molise, Contrada Fonte Lappone, 86090 Pesche, Italy; michele.innangi@unimol.it

3 Department of Chemical Sciences, University of Naples Federico II, Complesso Universitario Monte Sant'Angelo, Via Cintia 4, 80126 Napoli, Italy; jesus.zorrilla@uca.es (J.G.Z.); petraglia.gianmarco17@gmail.com (G.P.); mariateresa.russo2@unina.it (M.T.R.); alessio.cimmino@unina.it (A.C.)

4 Allelopathy Group, Department of Organic Chemistry, Facultad de Ciencias, Institute of Biomolecules (INBIO), University of Cadiz, C/Avenida República Saharaui, s/n, 11510 Puerto Real, Spain

* Correspondence: marco.masi@unina.it

Supporting information list

Figure S1. ^{1}H NMR spectrum of isoquercetin (**1**) (CD_3OD, 500 MHz).

Figure S2. ^{1}H NMR spectrum of rutin (**2**) (CD_3OD, 500 MHz).

Figure S3. ^{1}H NMR spectrum of isorhamnetin glucoside (**3**) (CD_3OD, 500 MHz).

Figure S4. ^{1}H NMR spectrum of narcissoside (**4**) (CD_3OD, 500 MHz).

Figure S5. ^{1}H NMR spectrum of quercetin malonylglucoside (**5**) (CD_3OD, 500 MHz).

Figure S6. ^{1}H NMR spectrum of isorhamnetin malonylglucoside (**6**) (CD_3OD, 500 MHz).

Figure S7. ESI MS spectrum of isoquercetin (**1**), recorded in negative mode.

Figure S8. ESI MS spectrum of rutin (**2**), recorded in negative mode.

Figure S9. ESI MS spectrum of isorhamnetin glucoside (**3**), recorded in negative mode.

Figure S10. ESI MS spectrum of narcissoside (**4**), recorded in negative mode.

Figure S11. ESI MS spectrum of quercetin malonylglucoside (**5**), recorded in negative mode.

Figure S12. ESI MS spectrum of isorhamnetin malonylglucoside (**6**), recorded in negative mode.

Figure S13. The HaCaT cells monolayer was scratched in the center with a sterile tip and treated with isoquercetin (**1**) or quercetin malonyl glucoside (**5**) at 50 μM, 75 μM, and 100 μM. Images were obtained immediately after the scratch (t0) at 5.5, 8, and 24 h after treatment.

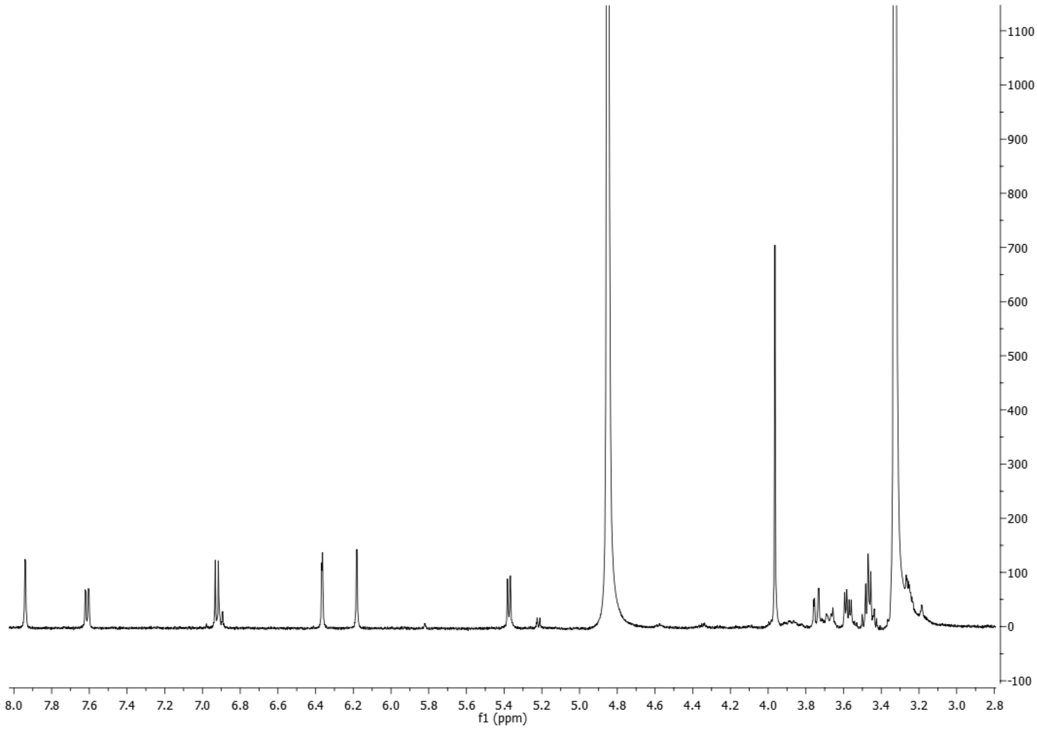

Figure S1. ^{1}H NMR spectrum of isoquercetin (**1**) (CD_3OD, 500 MHz).

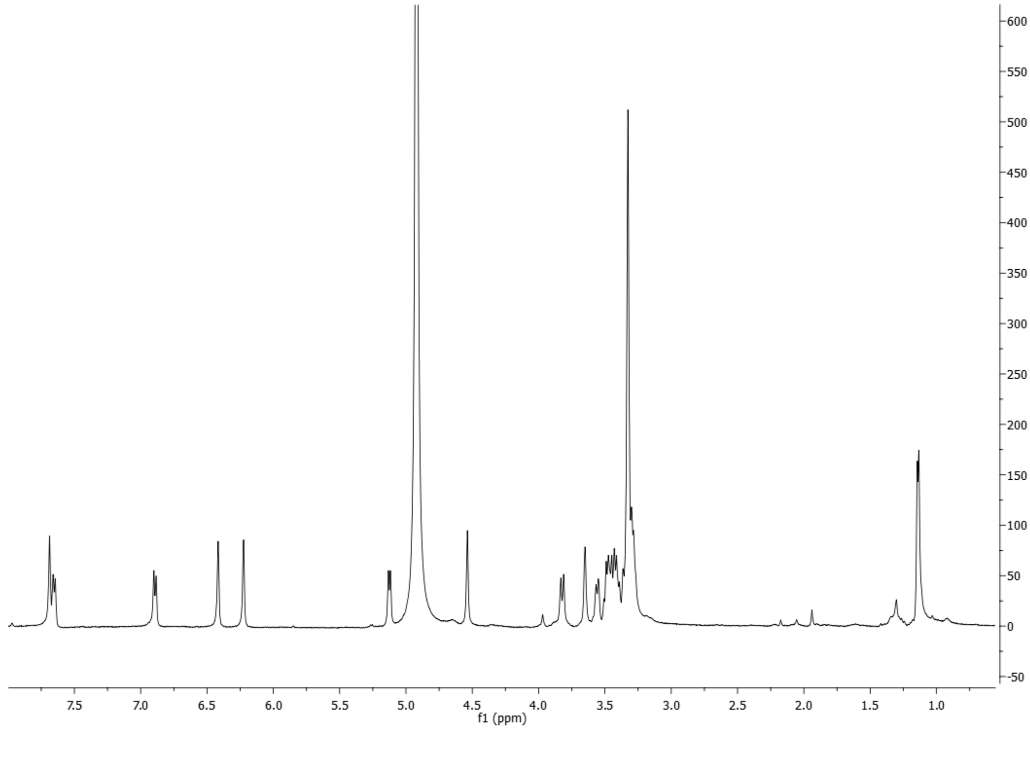

Figure S2. ^{1}H NMR spectrum of rutin (**2**) (CD_3OD, 500 MHz).

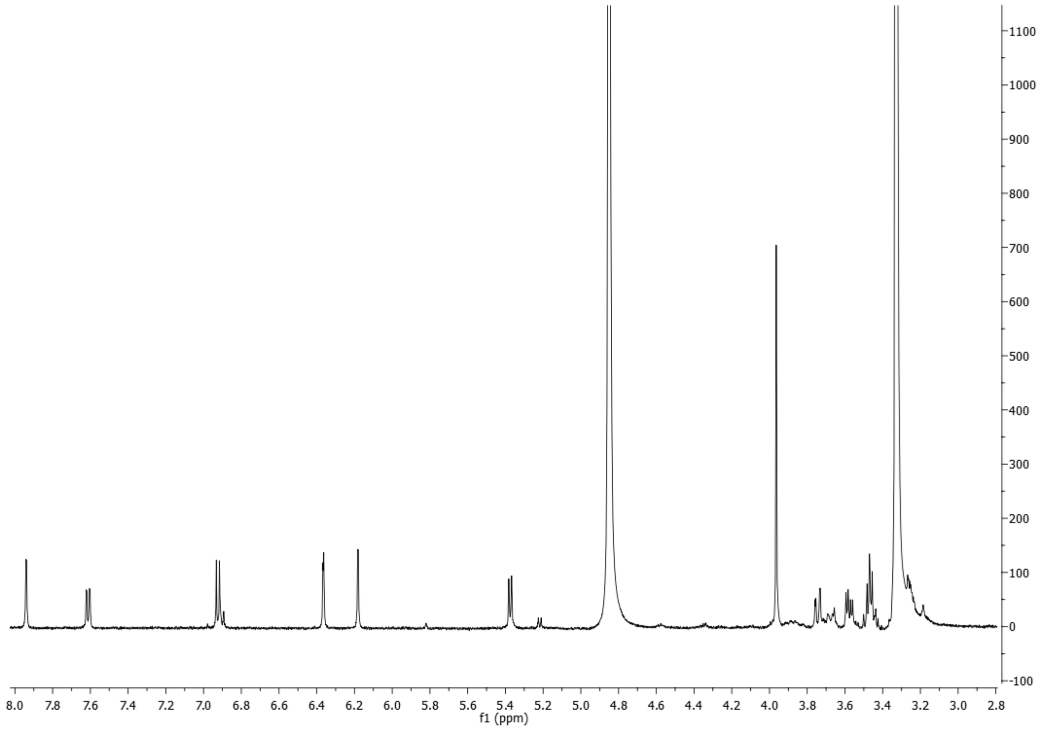

Figure S3. ^{1}H NMR spectrum of isorhamnetin glucoside (**3**) (CD_3OD, 500 MHz).

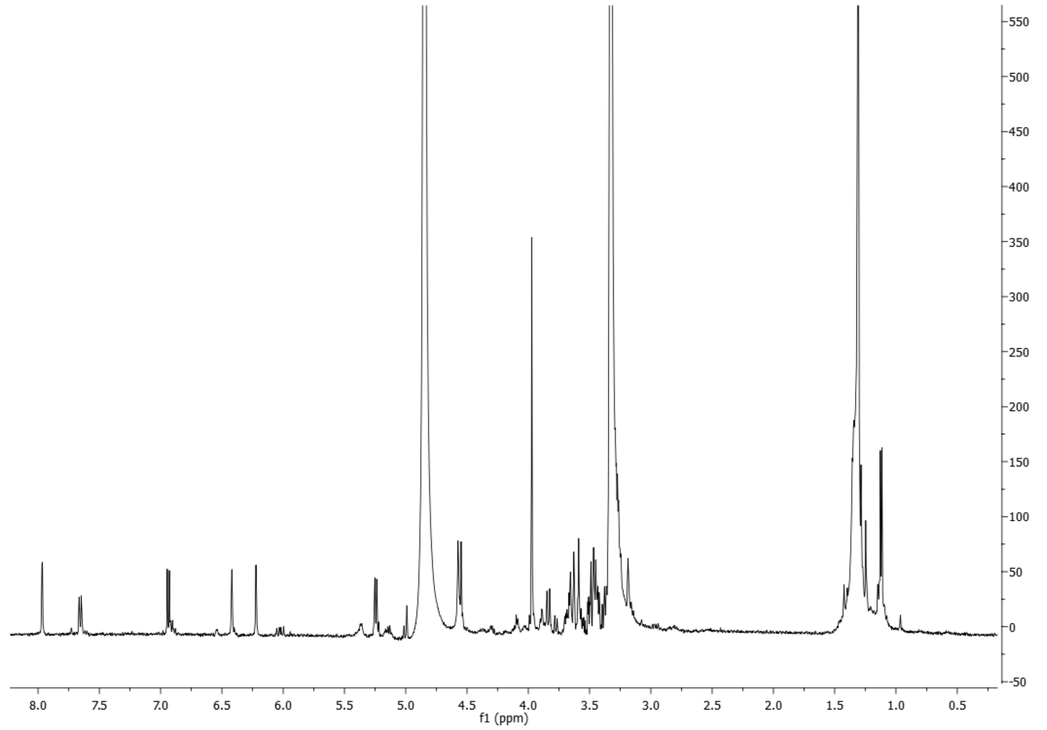

Figure S4. ^{1}H NMR spectrum of narcissoside (**4**) (CD_3OD, 500 MHz).

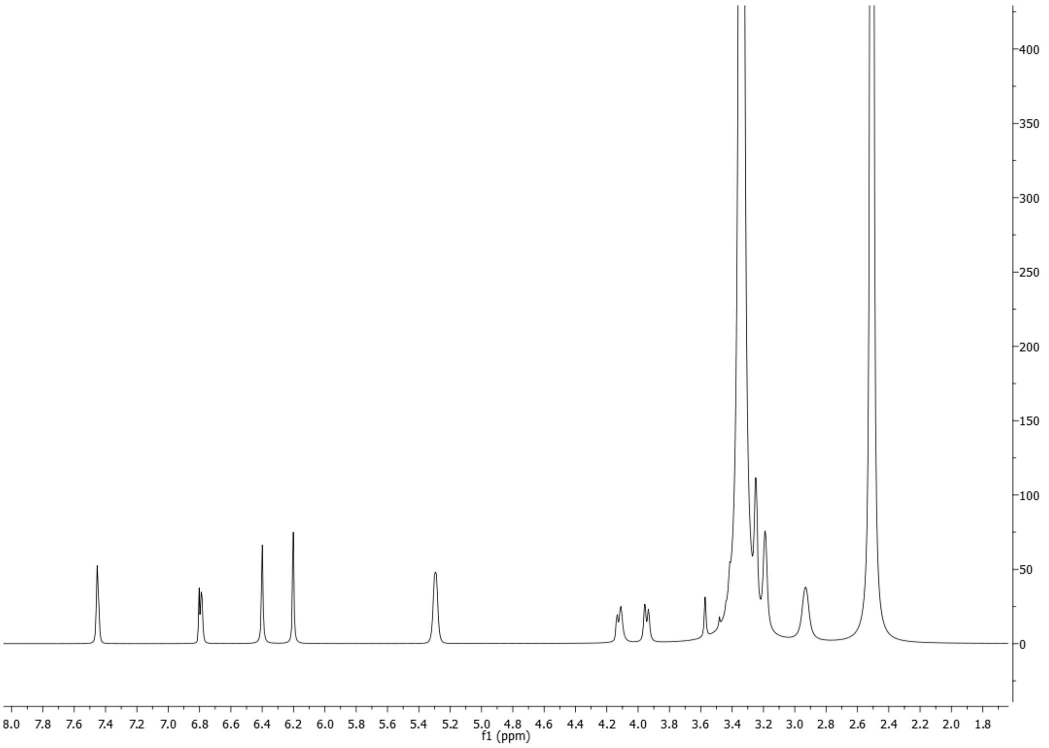

Figure S5. ^{1}H NMR spectrum of quercetin malonylglucoside (**5**) (CD_3OD, 500 MHz).

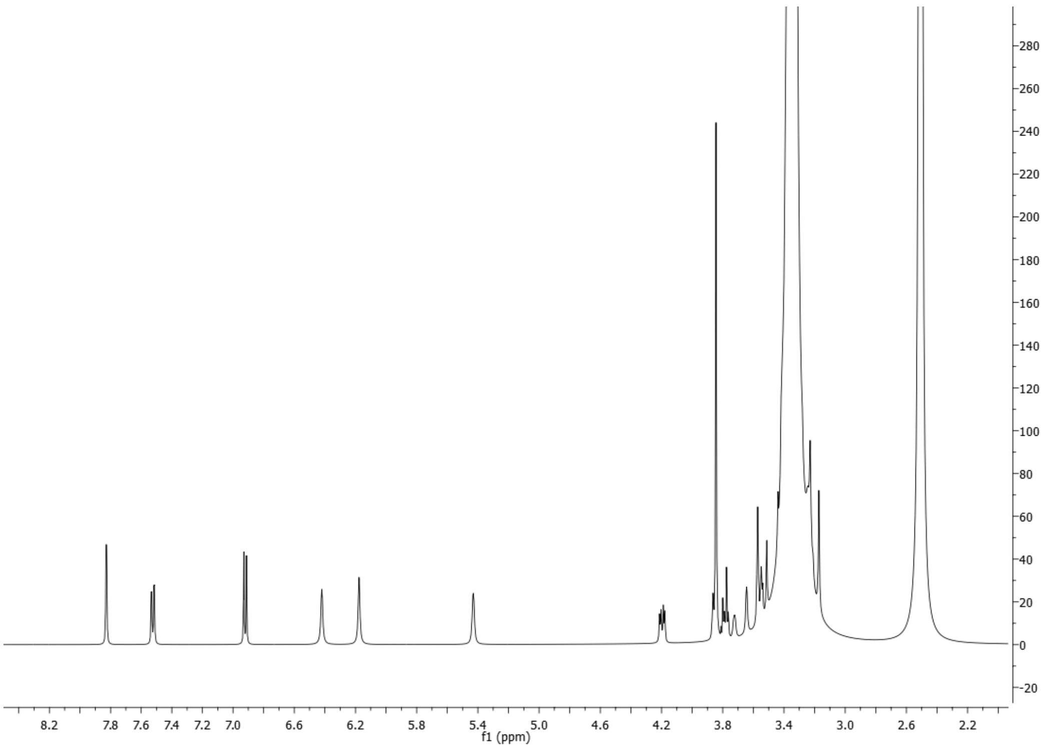

Figure S6. ^{1}H NMR spectrum of isorhamnetin malonylglucoside (**6**) (CD_3OD, 500 MHz).

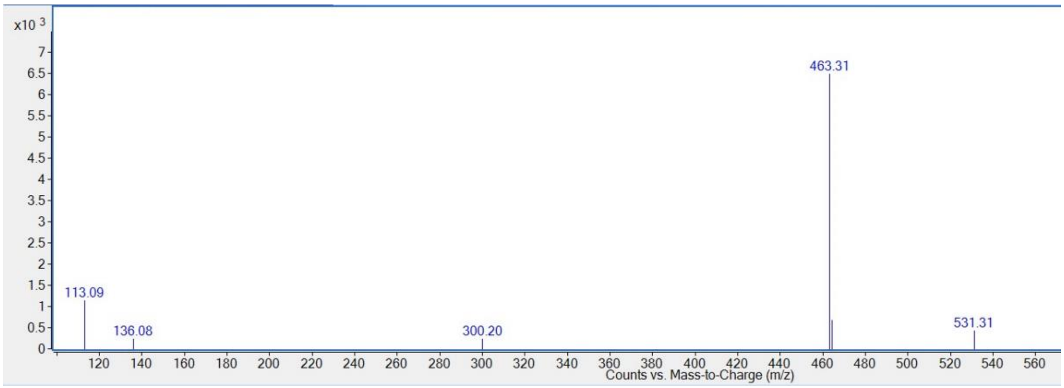

Figure S7. ESI MS spectrum of isoquercetin (**1**), recorded in negative mode.

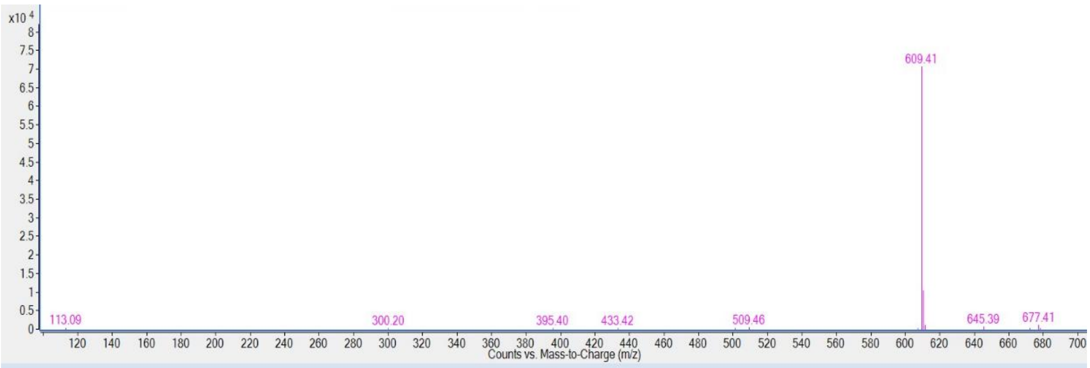

Figure S8. ESI MS spectrum of rutin (**2**) recorded in negative mode.

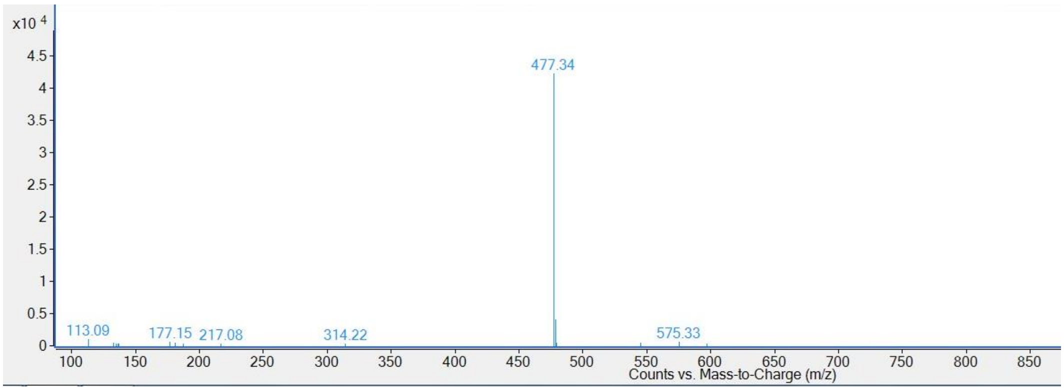

Figure S9. ESI MS spectrum of isorhamnetin glucoside (**3**), recorded in negative mode.

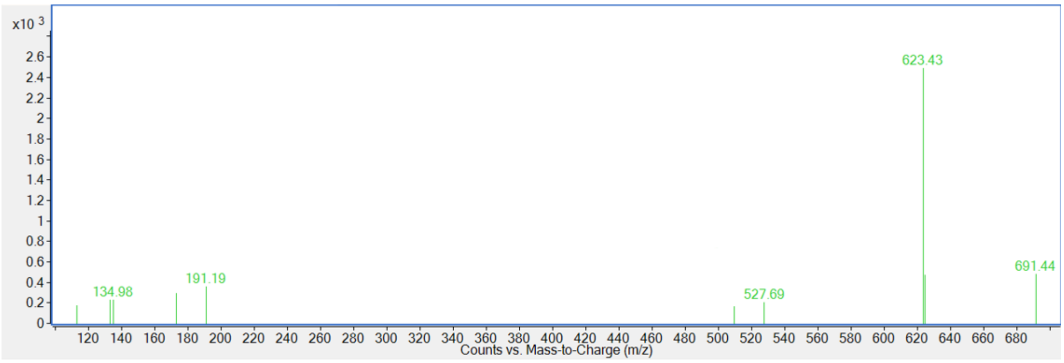

Figure S10. ESI MS spectrum of narcissoside (**4**), recorded in negative mode.

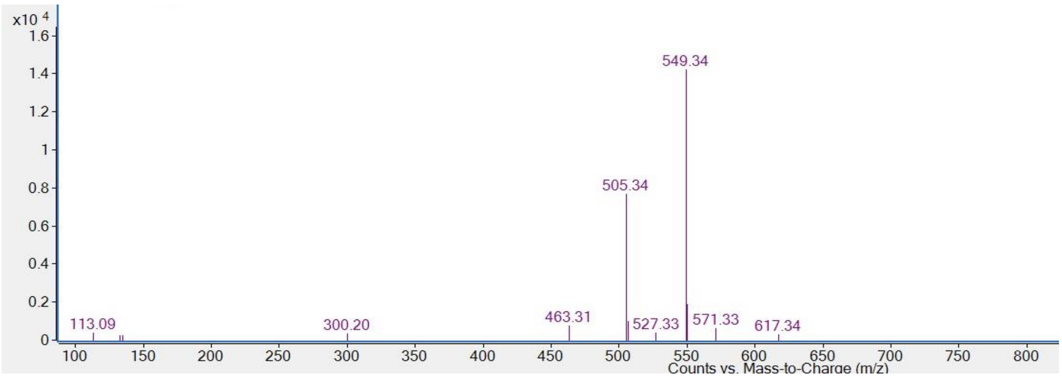

Figure S11. ESI MS spectrum of quercetin malonylglucoside (**5**), recorded in negative mode.

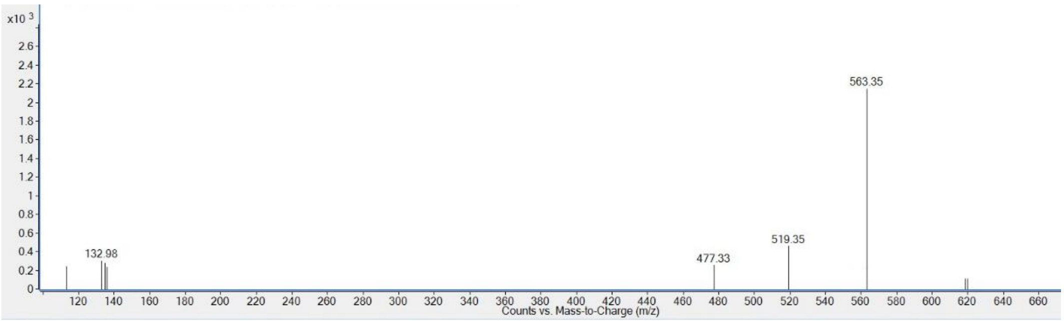

Figure S12. ESI MS spectrum of isorhamnetin malonylglucoside (**6**), recorded in negative mode.

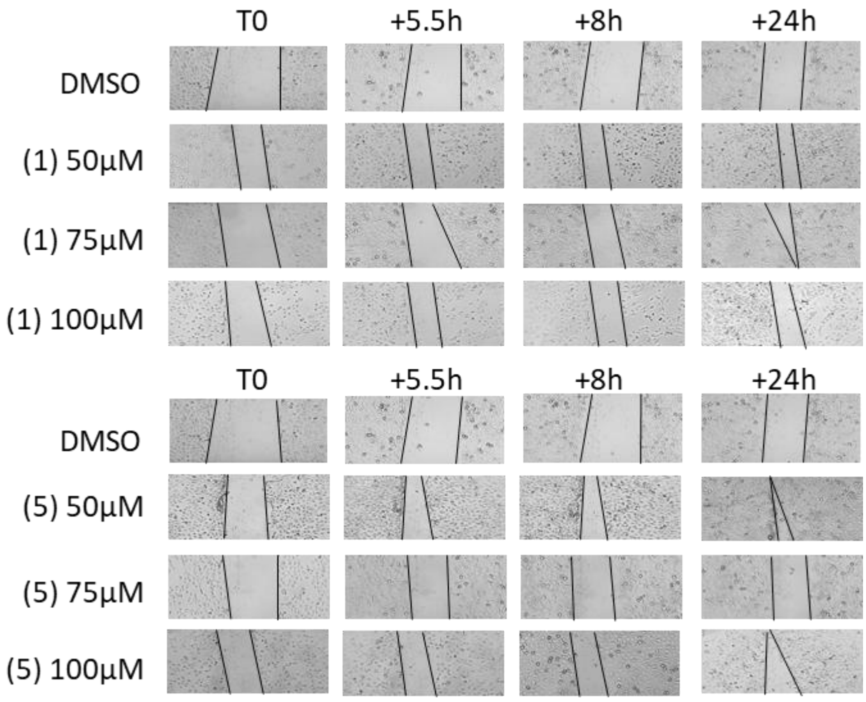

Figure S13. The HaCaT cells monolayer was scratched in the center with a sterile tip and treated with isoquercetin (**1**) or quercetin malonyl glucoside (**5**) at 50 µM, 75 µM, and 100 µM. Images were obtained immediately after the scratch (t0) at 5.5, 8, and 24 h after treatment.
